# Effects of mind–body exercise on knee osteoarthritis: a systematic review and meta-analysis of randomized controlled trials

**DOI:** 10.1186/s12891-024-07278-4

**Published:** 2024-03-22

**Authors:** Hujun Qiao, Xin Hao, Guoxiang Wang

**Affiliations:** 1 School of Physical Education, Soochow University, Suzhou, 215021 China; 2 Changzhi University, Changzhi, 046000 China

**Keywords:** Mind–body exercise, Taichi, Yoga, Baduanjin, Knee osteoarthritis, Meta-analysis

## Abstract

**Objective:**

To evaluate the effect of mind–body exercise on improving knee osteoarthritis (KOA) and thereby informing osteoarthritis exercise rehabilitation.

**Methods:**

The China National Knowledge Infrastructure (CNKI), Wanfang, PubMed/Medline, Cochrane Library, Web of Science, EBSCO, Embase, Scopus, and ProQuest databases were searched to identify randomized controlled trials (RCTs) that involved tai chi, yoga, and baduanjin interventions for KOA. The search period ranged from inception to October 25, 2022. The methodological quality of the included studies was evaluated by the Cochrane risk of bias assessment tool, and the included data were statistically analyzed and plotted using Review Manager 5.3 and Stata 14.0 software.

**Results:**

We included 17 articles with a total of 1122 patients. Compared with the control group, mind–body exercise significantly improved patient pain (standardized mean difference (SMD) = -0.65, 95% confidence interval (CI) [-0.87, -0.42], *p* < 0.00001), stiffness (SMD = -0.75, 95% CI [-1.05, -0.45], *p* < 0.00001), physical function (SMD = -0.82, 95% CI [-1.03, -0.62], *p* < 0.00001), mental health (SMD = 0.31, 95% CI [0.11, 0.51], *p* = 0.002), and depression (SMD = -0.32, 95% CI [-0.50, -0.15], *p* = 0.0003). In terms of motor ability, mind–body exercise significantly increased the 6-min walking distance (SMD = 18.45, 95% CI [5.80, 31.10], *p* = 0.004) and decreased timed up and go test time (SMD = -1.15, 95% CI [-1.71, -0.59], *p* < 0.0001).

**Conclusions:**

The current study showed that mind–body exercise is safe and effective for KOA patients. However, given the methodological limitations of the included studies, additional high-quality evidence is needed to support the conclusions of this study.

## Background

Knee osteoarthritis (KOA) is a common progressive joint disease involving tissues such as cartilage and subchondral bone; this disease can induce physical disability and functional impairment in elderly individuals [1]. Different factors cause KOA, such as aging, obesity, trauma, and genetics, which ultimately lead to problems such as joint pain, stiffness, and loss of joint function. The global incidence of (radiologically diagnosed) KOA is 3.8%, but among people older than 60 years, the incidence exceeds 10% [2]. At present, the clinical treatment goals are mostly limited to analgesia and improvement of function [3]. Except for surgical treatment (in cases when conservative treatment is ineffective), KOA can be treated by both drug and nondrug means [4]. While pharmacological interventions can reduce pain and improve physical function, accumulating evidence suggests that long-term use of drugs and intra-articular injections may cause adverse effects such as gastrointestinal reactions, multiple-organ failure, pain, and swelling [5]. Therefore, identification of simple and effective nondrug treatment methods is urgently needed.

In recent decades, complementary and alternative medicine has become popular among patients with various types of diseases [6]. Mind–body exercise has been incorporated into treatment and has been shown to improve neuromuscular activity and physical health [7]. Tai chi, yoga, and baduanjin are the three most popular forms of mind–body exercise; researchers have examined their effects on biological processes and responses such as inflammation [8]. Tai chi and baduanjin are traditional Chinese medicine (TCM) fitness exercises involving specific movements or postures, breathing coordination, and mental focus. Yoga originated in ancient India and usually includes specific body postures, breathing control, meditation, and relaxation. Accumulating evidence indicates that mind–body exercise can improve depressive symptoms [9], help in treating chronic obstructive pulmonary disease [10, 11], relieve chronic pain in middle-aged and elderly people [12], improve cognitive function in elderly individuals with mild cognitive impairment [13–15], and improve symptoms of posttraumatic stress disorder [16].

According to our review of KOA, few studies have integrated findings focused on different types of mind–body exercise as a whole. In the present study, we aimed to systematically evaluate and quantify the effects of mind–body exercise on pain, stiffness, physical function, mental health, depression, and motor ability in patients with KOA.

## Methods

### Registration and search strategy

This review was registered in the Prospective Register of Systematic Reviews (PROSPERO, ID: CRD42022367434). Articles were retrieved from nine electronic databases: China National Knowledge Infrastructure (CNKI), Wanfang, PubMed/Medline, Cochrane Library, Web of Science, EBSCO, Embase, Scopus, and ProQuest. In each database, we combined the following groups of terms for the search: (1) "Taichi" OR "Baduanjin" OR "Yoga"; (2) "osteoarthritis, knee" OR "KOA" OR "osteoarthritis of the knee". The period of the search ranged from database inception to October 25, 2022; relevant studies were added retrospectively.

### Study inclusion criteria

The included studies were all randomized controlled trials (RCTs) published in Chinese or English. The subjects were patients with confirmed KOA. The interventions conducted in the experimental group included tai chi, yoga, or baduanjin, and the intervention duration was at least 8 weeks. The control group interventions involved health education, physical therapy, attentional control, or no intervention. The outcome indicators were pain, stiffness, physical function, mental health, depression, timed up and go (TUG) test time, and 6-min walk test (6-MWT) time.

### Study exclusion criteria

The exclusion criteria for studies were as follows: 1) the experimental group underwent mind–body exercise combined with other interventions, 2) the study lacked a control group or had a control group involving another exercise intervention, 3) data related to outcome indicators could not be extracted, 4) duplicate publications, or 5) the full text could not be obtained.

### Study screening and data extraction

The data extraction form was designed in advance, and information extraction was completed independently by two authors. The extracted information included the following: 1) basic study information (first author, publication year, country, and research design), 2) characteristics of the research subjects (age, sex, diagnostic method, and sample size), 3) experimental intervention (exercise form, exercise frequency, and exercise cycle), 4) control conditions, 5) outcome indicators, 6) adverse reactions, and 7) relevant information regarding study quality. The above two authors resolved any disagreements through discussion, and the third author provided suggestions when the disagreement could still not be resolved.

We compared the differences in the changes between the intervention and control groups to determine the effects of mind–body exercise. The data are expressed as the means and standard deviations (SDs), and the data in other formats (such as 95% confidence intervals (CIs)) were translated to the means ± SDs as described in the Cochrane Handbook [17].

### Evaluation of study quality

The methodological quality of the included studies was assessed using the Cochrane risk of bias assessment tool. The assessed items included random sequence generation, allocation sequence concealment, blinding of subjects and investigators, blinding of outcome measurers, outcome data incompleteness, selective reporting, and other potential sources of bias. According to the risk of bias assessment criteria, a judgment of "low risk of bias", "high risk of bias", or "unclear" was issued for each item.

### Statistical analysis

This study used Review Manager 5.3 statistical software for study quality evaluation, data merging, heterogeneity testing, and forest plot generation. The extracted outcome indicators were all continuous variables. If the measurement tools used were the same among the studies, the weighted mean difference (WMD) and 95% CI were selected as the effect magnitude for analysis; if the measurement tools were different, the standard mean difference (SMD) and its 95% CI were used as the effect magnitude for analysis.

Heterogeneity was evaluated with I^2^ values: I^2^ ≤ 25% indicated mild heterogeneity; 25% < I^2^ < 50% indicated moderate heterogeneity; 50% < I^2^ < 75% indicated substantial heterogeneity; and I^2^ ≥ 75% indicated high heterogeneity. When I^2^ < 50% and *p* ≥ 0.1, a fixed-effects model was used for meta-analysis; otherwise, a random-effects model was used for analysis. When the merged data included more than 10 items and the heterogeneity was substantial, subgroup analysis, Egger's test and sensitivity analysis were performed with Review Manager 5.3 and Stata 14.0.

## Results

### Literature search results

**Fig. 1:**
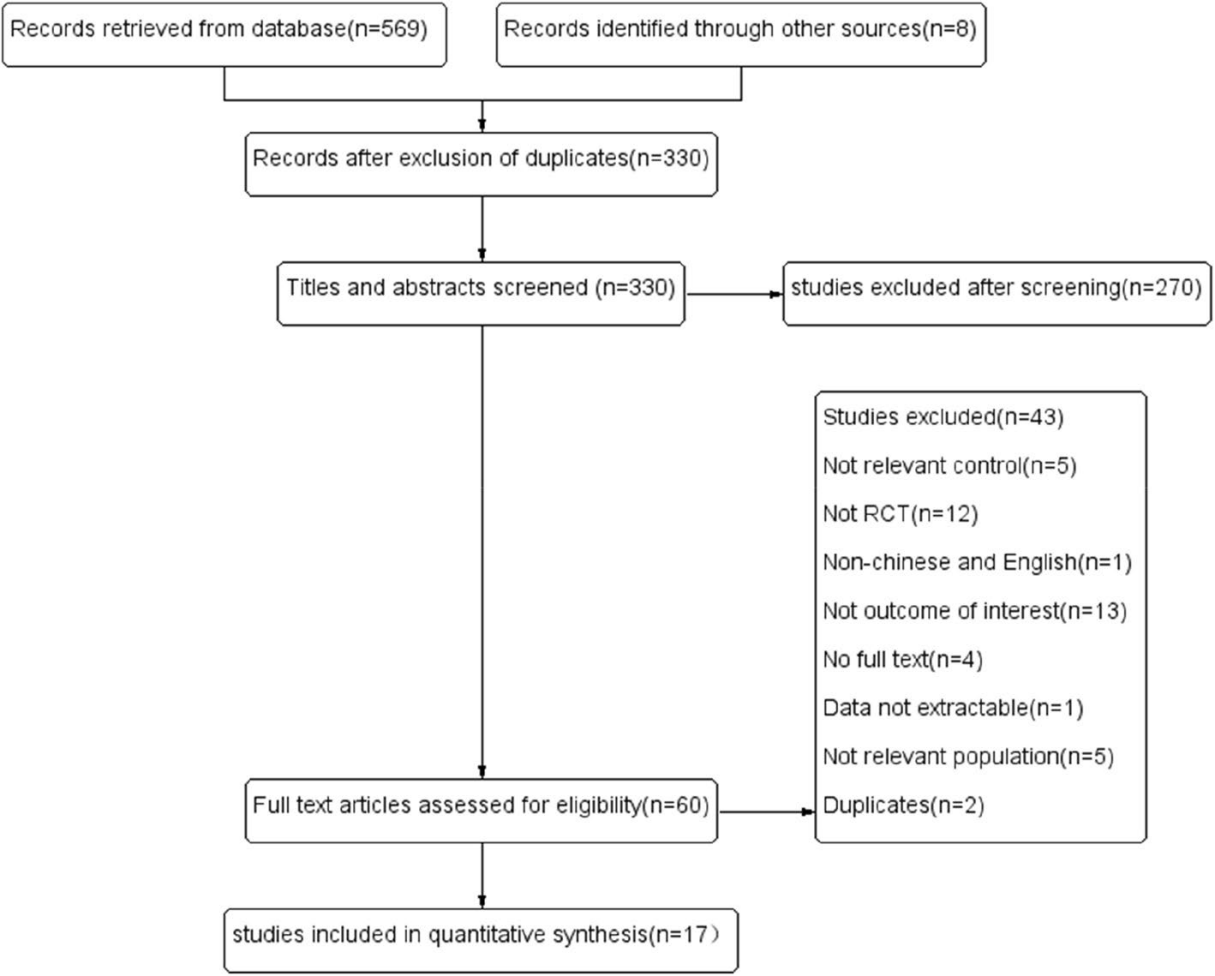
PRISMA flow chart of articles screening procedure

The preliminary search yielded 569 articles in the searched databases, including the CNKI (8 articles), Wanfang (36 articles), PubMed/Medline (18 articles), Cochrane Library (55 articles), Web of Science (93 articles), EBSCO (46 articles), Embase (160 articles), Scopus (136 articles), and ProQuest databases (17 articles). Eight supplementary articles were retrospectively added, for a total of 577 articles. The bibliographies was imported into EndNote X7, and 330 articles remained after duplicates were eliminated. After screening, 17 articles were ultimately included in the quantitative analysis (Fig. 1).

### Study characteristics

The basic characteristics of the included studies are shown in Table 1 and 2. A total of 17 articles (18 studies) were included in this study, including two studies extracted from the paper of Liu et al. [18], with a total sample of 1122 people. The studies were published from 2008 to 2022 and were conducted in China (*n* = 7), Australia (*n* = 1), the USA (*n* = 7), Canada (*n* = 1), or Korea (*n* = 1). Participants were diagnosed with KOA by the American College of Rheumatology (ACR) classification criteria, the diagnostic criteria of the American Rheumatism Association (ARA), the Kellgren-Lawrence Scale (KL), the National Institute for Health and Care Excellence (NICE) osteoarthritis clinical criteria, X-ray imaging, or physician confirmation.

**Table 1:** Basic information of the included studies

Reference	Country	Diagnostic Criteria	Study Design	Female(%)		Sample Size		Age (Mean ± SD or Range)(Years)		BMI(kg/m^2^)	
				**Exp**	**Ctrl**	**Exp**	**Ctrl**	**Exp**	**Ctrl**	**Exp**	**Ctrl**
An B et al., 2008 [19]	China	ACR	2-arm	100%	100%	11	10	65.4 ± 8.2	64.6 ± 6.7	25.7 ± 2.9	25.4 ± 2.9
Brismeé et al., 2007 [20]	USA	ARA	2-arm	86.4%	78.9%	18	13	70.9 ± 9.8	68.9 ± 8.9	28 ± 5.9	27.8 ± 6.6
Bennell K L et al., 2022 [21]	Australia	NICE	2-arm	65.4%	74.8%	105	107	62.8 ± 8.2	61.8 ± 7.2	29.9 ± 4.8	30.7 ± 6.1
Chenchen W et al., 2016 [22]	USA	ACR	2-arm	71%	69%	106	98	60.3 ± 10.5	60.1 ± 10.5	33.0 ± 7.1	32.6 ± 7.3
Cheung C et al., 2014 [23]	USA	ACR	2-arm	100%	100%	18	18	71.9 ± 5.33	71.9 ± 6.0	29.1 ± 5.0	28.8 ± 5.7
Cheung C et al., 2017 [24]	USA	ACR	3-arm	84%		32	23	68.9 ± 7.7	71.8 ± 8.0	29.8 ± 6.3	27.8 ± 7.9
Hu X Y et al., 2020 [25]	China	X-ray	2-arm	100%	100%	52	40	66.3 ± 4.2	65.5 ± 3.6	36.5 ± 9.0	26.4 ± 3.1
Kuntz A B et al., 2018 [26]	Canada	ACR	3-arm	100%	100%	10	10	65.5 ± 5.6	71.1 ± 9.3	30.1 ± 3.8	32.3 ± 5.7
Lee H J et al., 2009 [27]	Korea	KL scale	2-arm	93.1%	93.3%	29	15	70.2 ± 4.8	66.9 ± 6.0	26.0 ± 3.8	26.0 ± 2.8
Jingya L et al., 2019 [28]	China	ACR	3-arm	38.7%	36.7%	31	30	65.8 ± 6.7	66.0 ± 5.0	/	/
Liu J et al., 2019A [18]	China	ACR	4-arm	78.6%	58.3%	28	24	40–70	40–70	22.8 ± 2.2	23.4 ± 3.3
Liu J et al., 2019B [18]	China	ACR	4-arm	82.8%	58.3%	29	24	40–68	40–70	23.1 ± 2.7	23.4 ± 3.3
Tsai P-F et al., 2013 [29]	USA	health care provider	2-arm	78.6%	66.7%	28	27	78.9 ± 6.9	78.9 ± 8.3	/	/
Wang C et al., 2009 [30]	USA	ACR	2-arm	80%	70%	20	20	63 ± 8.1	68 ± 7.0	30.0 ± 5.2	29.8 ± 4.3
Wortley M et al., 2013 [31]	USA	KL scale	3-arm	75%	66.7%	12	6	68.1 ± 5.3	70.5 ± 5.0	35.1 ± 5.9	30.0 ± 6.2
Ye J et al., 2019 [32]	China	ACR	2-arm	52%	68%	25	25	64.5 ± 7.8	63.1 ± 3.65	24.2 ± 2.5	24.6 ± 2.3
Ye J et al., 2020 [33]	China	ACR	2-arm	60.7%	71.4%	28	28	65.1 ± 6.6	63.6 ± 2.6	24.2 ± 2.4	24.6 ± 2.3
Zhu Q et al., 2016 [34]	China	ACR	2-arm	100%	100%	23	23	64.6 ± 3.4	64.5 ± 3.4	25.2 ± 3.5	25.1 ± 3.4

*Exp* Experimental group, *Ctrl* Control group, *ACR* American College of Rheumatology, *ARA* American Rheumatism Association, *KL* Kellgren–Lawrence Scale, *2-arm* Two-arm experiment, *3-arm* Three-arm experiment, *4-arm* Four-arm experiment, *NICE* National Institute for Health and Care Excellence

**Table 2:** Mind–body exercise Intervention and Outcome Index of the Included Studies

**Reference**	Exp			Ctrl			Time-point	Outcomes Measure	Adverse Effects
	Intervention	Duration	Frequency	Intervention	Duration	Frequency			
An B et al., 2008 [19]	Baduanjin	30 min	5	/	/	/	8 weeks		No adverse event
Brismeé et al., 2007 [20]	Tai Chi	40 min	3	Attention control	/	/	12 weeks		No adverse event
Bennell K L et al., 2022 [21]	Yoga	30nin	3	Healthy education	/	/	12 weeks		No adverse event
Chenchen W et al., 2016 [22]	Tai Chi	60 min	2	Physical therapy	30 min	2–4	12 weeks		No adverse event
Cheung C et al., 2014 [23]	Yoga	30/60 min	5	/	/	/	8 weeks		No adverse event
Cheung C et al., 2017 [24]	Yoga	30/45 min	5	Healthy education	/	/	8 weeks		No adverse event
Hu X Y et al., 2020 [25]	Tai Chi	60 min	3	Healthy education	/	/	24 weeks		/
Kuntz A B et al., 2018 [26]	Yoga	60 min	3	Meditation	60 min	3	12 weeks		No adverse event
Lee H J et al., 2009 [27]	Tai Chi	60 min	2	/	/	/	8 weeks		No adverse event
Jingya L et al., 2019 [28]	Tai Chi	60 min	4	/	/	/	16 weeks		/
Liu J et al., 2019A [18]	Tai Chi	60 min	5	Healthy education	60 min	1	12 weeks		No adverse event
Liu J et al., 2019B [18]	Baduanjin	60 min	5	Healthy education	60 min	1	12 weeks		No adverse event
Tsai P-F et al., 2013 [29]	Tai Chi	20/40 min	3	Healthy education	/	/	20 weeks		No adverse event
Wang C et al., 2009 [30]	Tai Chi	60 min	2	Attention control	60 min	2	12 weeks		No adverse event
Wortley M et al., 2013 [31]	Tai Chi	60 min	2	/	/	/	10 weeks		No adverse event
Ye J et al., 2019 [32]	Baduanjin	40 min	3	/	/	/	12 weeks		No adverse event
Ye J et al., 2020 [33]	Baduanjin	40 min	3	/	/	/	12 weeks		No adverse event
Zhu Q et al., 2016 [34]	Tai Chi	60 min	3	Healthy education	60 min	1	24 weeks		No adverse event

Outcomes: Pain, Stiffness, Physical function, Mental health, Depression, 6-Minute Walk Test, Timed Up and Go test

Of the 18 included studies, 10 studies [18, 20, 22, 25, 27–31, 34] involved tai chi as the intervention, four involved yoga [21, 23, 24, 26], and four involved baduanjin [18, 19, 32, 33]. The duration of the mind–body exercises ranged from 8 to 24 weeks, with 2–5 exercise sessions per week. There were almost no reports of adverse events related to mind–body exercises.

### Risk of bias assessment

**Fig. 2:**
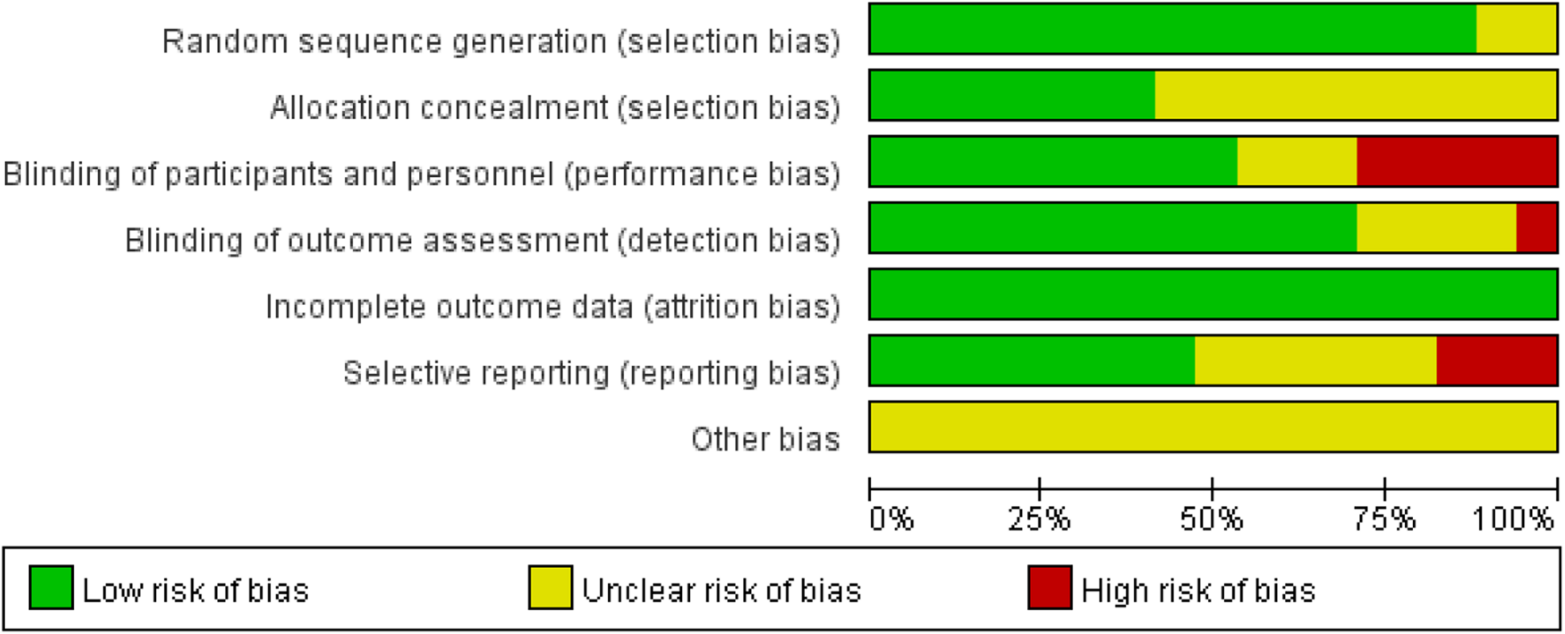
Risk of bias graph

**Fig. 3:**
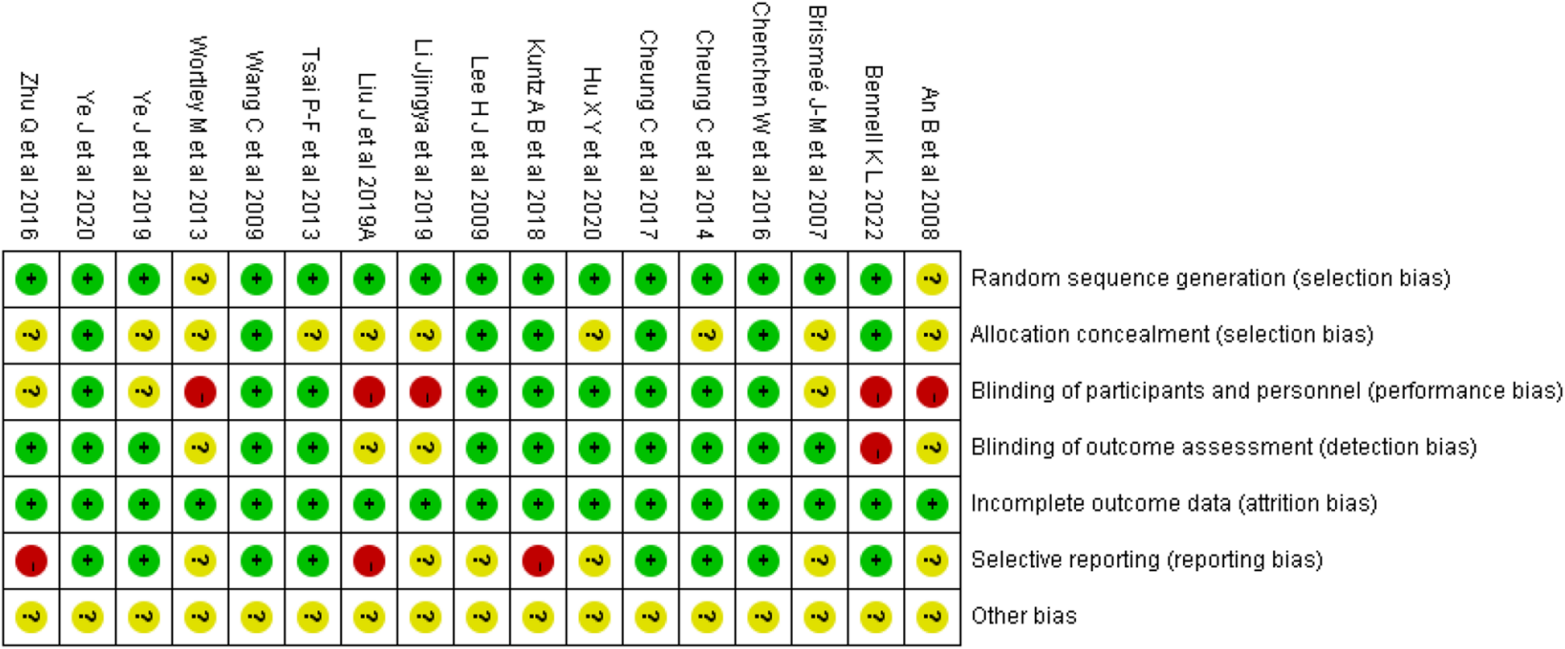
Risk of bias summary

Of the 17 articles included, 15 [18, 20–30, 32–34] described the process of generating random sequences, seven [21, 22, 24, 26, 27, 30, 33] reported allocation concealment, and 12 [20, 22–27, 29, 30, 32–34] reported the blinding of outcome assessors. Among the included articles, 5 [18, 19, 21, 28, 31] had a high risk of performance bias because they failed to blind the participants and personnel. One article [21] reported the outcomes by using online questionnaires (self-reported) and thus was considered to have a high risk of detection bias. Three articles [18, 26, 34] did not report all outcomes and so were categorized as having a high risk of reporting bias. The risk assessment results of all included studies are shown in Figs. 2 and 3.

### Data synthesis analysis

#### Effects of mind–body exercise on pain, stiffness, and physical function indicators

In the included studies, pain, stiffness, and physical function were measured using the Western Ontario and McMaster Universities Osteoarthritis Index (WOMAC) or the Knee Injury and Osteoarthritis Outcome Score (KOOS). Due to the use of different measurement tools, we calculated the SMDs and their 95% CIs to determine the effect sizes of the interventions.

**Fig. 4:**
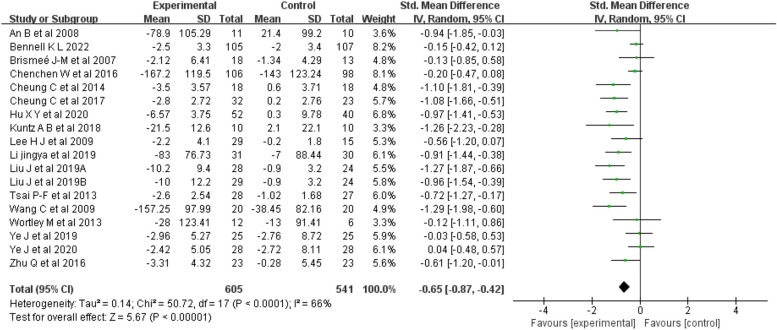
Forest plots showing standardized mean difference of change on the pain between Mind–body exercise group and a control/comparison group

Pain: Seventeen articles [18–34] (18 studies) were included in the pain analysis, for a total sample of 1122 people. The results indicated (Fig. 4) that mind–body exercise was significantly better than was the control intervention at improving pain (random-effects model, SMD = -0.65; 95% CI = -0.87, -0.42; *p* < 0.00001).

The meta-analysis showed that there was substantial heterogeneity between studies (I^2^ = 66%, *p* < 0.0001). Subgroup analysis was performed to explore the possible sources of heterogeneity and showed that duration, frequency, and sex were significant factors influencing the heterogeneity of studies on pain (Table 3).

**Table 3:** Results of subgroup analysis affecting pain heterogeneity

Groups	No. of studies	No. of Participants	SMD[95%CI]	Heterogeneity		*P*
				P	I^2^	
Exercise type						
Taichi	10	643	-0.69 [-0.97, -0.41]	0.005	62%	0.000
Yoga	4	323	-0.83 [-1.48, -0.18]	0.002	80%	0.002
Baduanjin	4	180	-0.42 [-0.97, 0.12]	0.02	68%	0.13
Duration time						
< 12 weeks	5	174	-0.83 [-1.15, -0.51]	0.4	1%	0.000
12 weeks	9	718	-0.51 [-0.84, -0.18]	0.000	74%	0.002
> 12 weeks	4	254	-0.83 [-1.09, -0.57]	0.76	0%	0.000
Frequency						
2 sessions per week	4	306	-0.53 [-1.04, -0.02]	0.03	66%	0.04
3 sessions per week	8	562	-0.43 [-0.74, -0.12]	0.007	64%	0.006
4–5 sessions per week	6	278	-1.04 [-1.30, -0.79]	0.97	0%	0.000
Gender						
Females	5	215	-0.93 [-1.21, -0.64]	0.77	0%	0.000
Females/males	13	931	-0.55 [-0.81, -0.29]	0.000	69%	0.000
Region						
Asia	9	475	-0.68 [-0.98, -0.37]	0.01	60%	0.000
Non-Asia	9	671	-0.62 [-0.94, -0.29]	0.001	69%	0.000

**Fig. 5:**
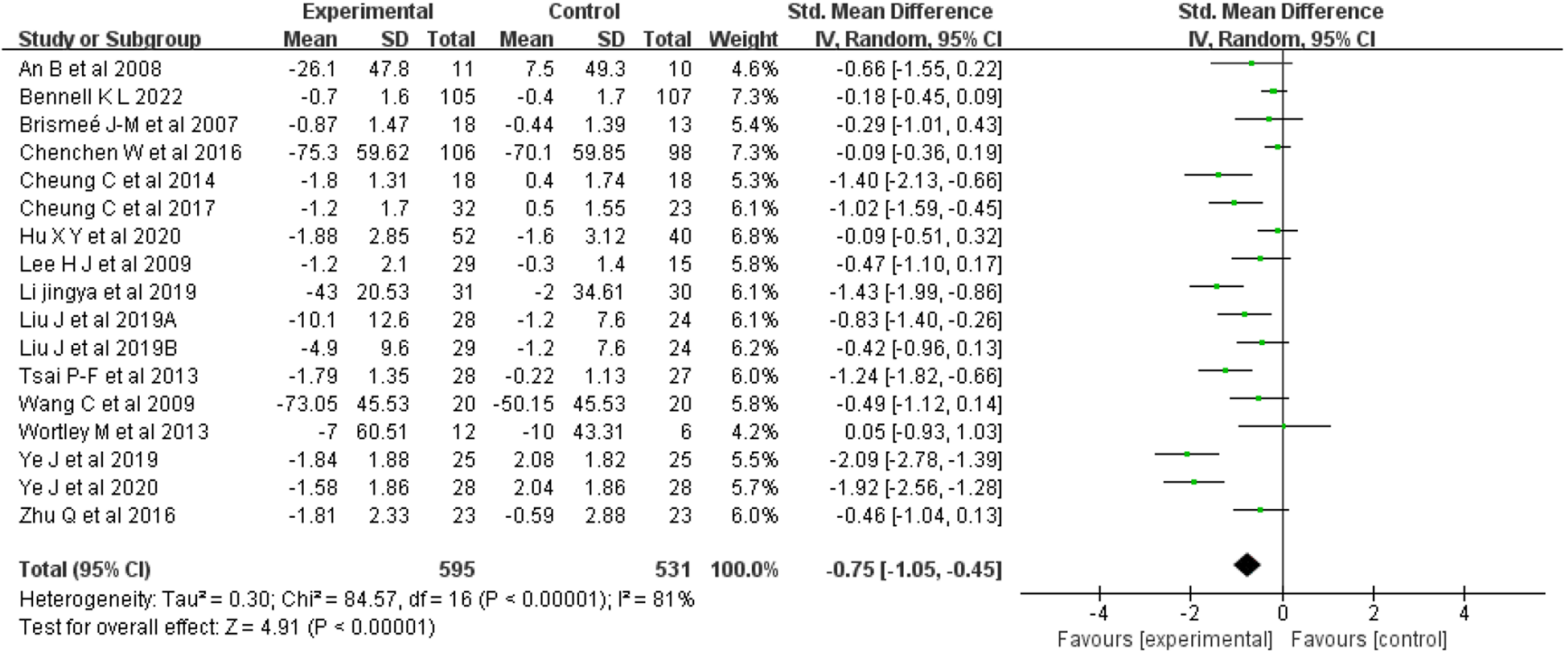
Forest plots showing standardized mean difference of change on the stiffness between Mind–body exercise group and a control/comparison group

Stiffness: Sixteen articles [18–25, 27–34] (17 studies) were included in the stiffness analysis, for a total sample of 1102 people. The results indicated (Fig. 5) that mind–body exercise was significantly better than was the control condition at improving stiffness (random-effects model, SMD = -0.75; 95% CI: -1.05, -0.45; *p* < 0.00001).

The meta-analysis showed that there was high heterogeneity among the studies (I^2^ = 81%, *p* < 0.00001). The subgroup analysis showed that duration and frequency were significant factors influencing the heterogeneity of studies on stiffness (Table 4).

**Table 4:** Results of subgroup analysis affecting stiffness heterogeneity

Groups	No. of studies	No. of Participants	SMD[95%CI]	Heterogeneity		*P*
				P	I^2^	
Exercise type						
Taichi	10	643	-0.54 [-0.86, -0.22]	0.000	71%	0.001
Yoga	3	303	-0.81 [-1.59, -0.03]	0.000	86%	0.04
Baduanjin	4	180	-1.27 [-2.16, -0.39]	0.001	85%	0.005
Duration time						
< 12 weeks	5	174	-0.76 [-1.20, -0.32]	0.13	44%	0.000
12 weeks	8	698	-0.75 [-1.21, -0.28]	0.000	87%	0.002
> 12 weeks	4	254	-0.79 [-1.45, -0.13]	0.000	84%	0.02
Frequency						
2 sessions per week	4	306	-0.18 [-0.41, 0.05]	0.49	0%	0.12
3 sessions per week	7	542	-0.87 [-1.44, -0.29]	0.000	89%	0.003
4–5 sessions per week	6	278	-0.96 [-1.29, -0.62]	0.13	41%	0.000
Gender						
Females	4	195	-0.60 [-1.15, -0.04]	0.02	68%	0.04
Females/males	13	931	-0.79 [-1.15, -0.43]	0.000	84%	0.000
Region						
Asia	9	475	-0.91 [-1.38, -0.44]	0.000	82%	0.000
Non-Asia	8	651	-0.56 [-0.92, -0.20]	0.000	75%	0.002

**Fig. 6:**
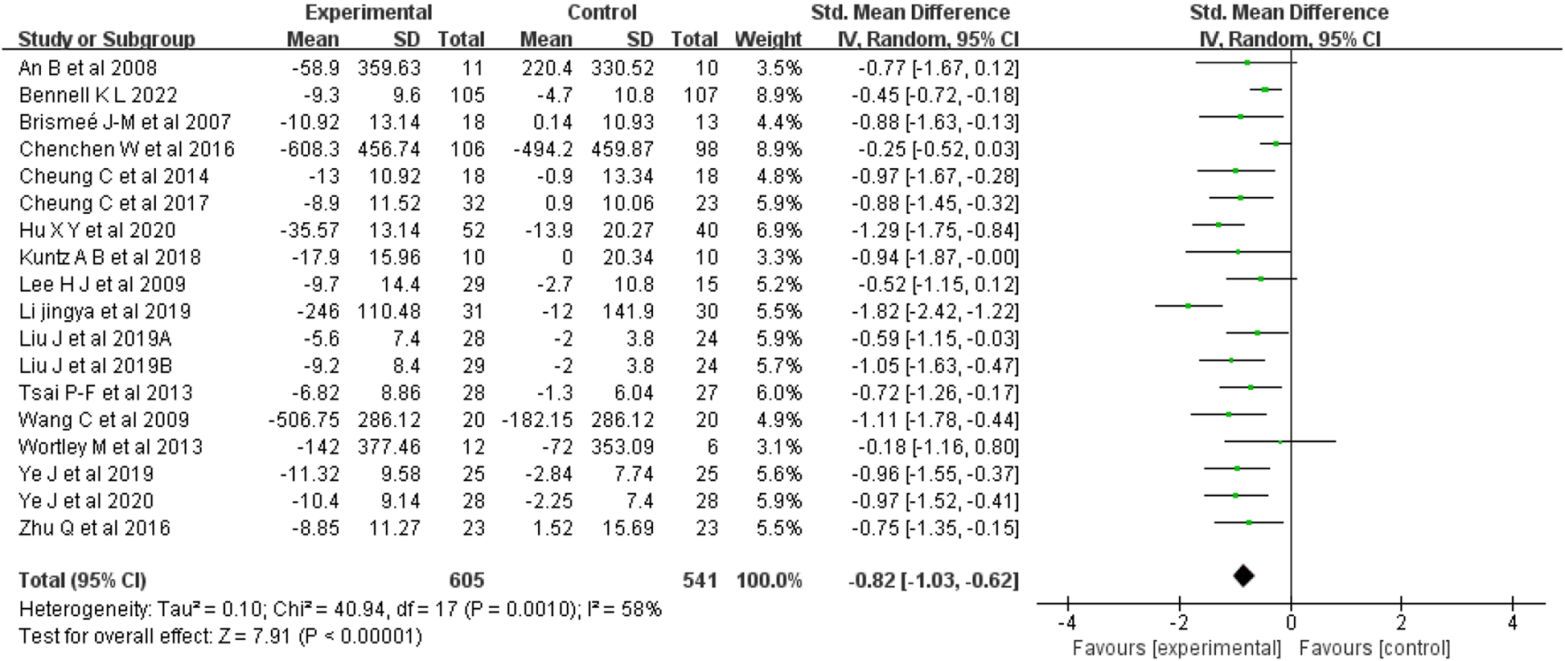
Forest plots showing standardized mean difference of change on the physical function between Mind–body exercise group and a control/comparison group

Physical function: Seventeen articles [18–34] (18 studies) were included in the physical function analysis, for a total sample of 1122 people. The results suggested (Fig. 6) that mind–body exercise was significantly better than was the control condition at improving physical function (random-effects model, SMD = -0.82; 95% CI: -1.03, -0.62; *p* < 0.00001).

The meta-analysis showed that there was substantial heterogeneity among the studies (I^2^ = 58%, *p* = 0.001). The subgroup analysis showed that exercise type, duration, frequency, sex, and region were significant factors influencing the heterogeneity of studies on physical function (Table 5).

**Table 5:** Results of subgroup analysis affecting physical function heterogeneity

Groups	No. of studies	No. of Participants	SMD[95%CI]	Heterogeneity		*P*
				P	I^2^	
Exercise type						
Taichi	10	643	-0.82 [-1.16, -0.48]	0.000	73%	0.000
Yoga	4	323	-0.66 [-0.95, -0.37]	0.29	20%	0.000
Baduanjin	4	180	-0.97 [-1.28, -0.66]	0.97	0%	0.000
Duration time						
< 12 weeks	5	174	-0.72 [-1.04, -0.41]	0.66	0%	0.000
12 weeks	9	718	-0.71 [-0.95, -0.47]	0.04	50%	0.000
> 12 weeks	4	254	-1.14 [-1.62, -0.66]	0.03	68%	0.000
Frequency						
2 sessions per week	4	306	-0.49 [-0.89, -0.08]	0.12	48%	0.02
3 sessions per week	8	562	-0.83 [-1.07, -0.59]	0.12	39%	0.000
4–5 sessions per week	6	278	-1.03 [-1.38, -0.67]	0.09	48%	0.000
Gender						
Females	5	215	-1.03 [-1.31, -0.74]	0.64	0%	0.000
Females/males	13	931	-0.78 [-1.02, -0.54]	0.001	64%	0.000
Region						
Asia	9	475	-0.99 [-1.25, -0.73]	0.08	43%	0.000
Non-Asia	9	671	-0.62 [-0.85, -0.40]	0.12	37%	0.000

### Effect of mind–body exercise on mental health and depression indicators

In the included studies, the 12-item Short Form Survey (SF-12) and SF-36 were used to assess mental health. Depression was measured with scales such as the Beck Depression Inventory, Hospital Anxiety and Depression Scale, and Center for Epidemiologic Studies Depression Scale. Due to the differences in the measurement tools used, the effect sizes of the included studies were calculated according to the SMDs and 95% CIs.

**Fig. 7:**
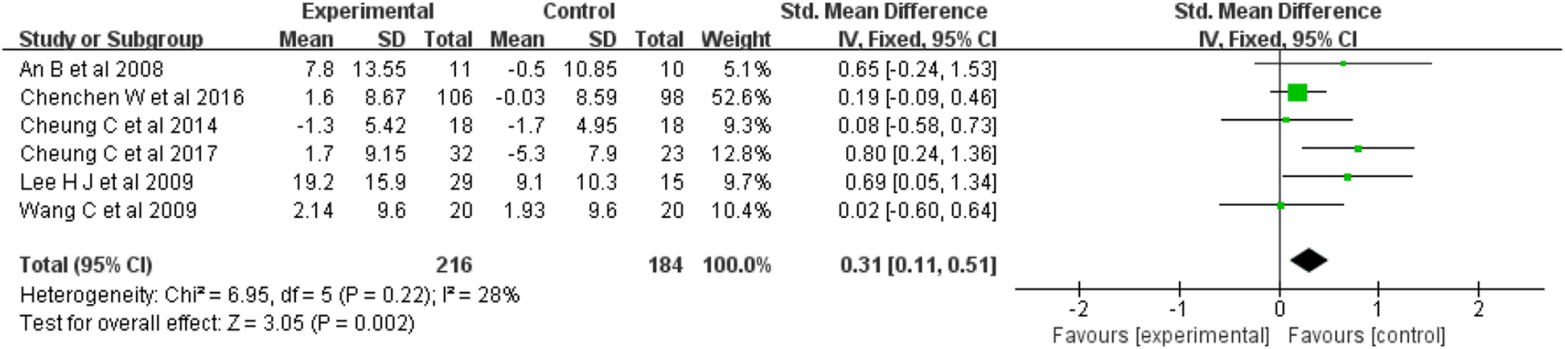
Forest plots showing standardized mean difference of change on the mental health between Mind–body exercise group and a control/comparison group

Mental Health: Six studies [19, 22–24, 27, 30] were included in the analysis, for a total sample of 400 people. The meta-analysis indicated moderate heterogeneity among the studies (I^2^ = 28%, *p* = 0.22). The results indicated (Fig. 7) that mind–body exercise was significantly better than was the control condition at improving mental health (fixed-effects model, SMD = 0.31; 95% CI = 0.11, 0.51; *p* = 0.002).

**Fig. 8:**
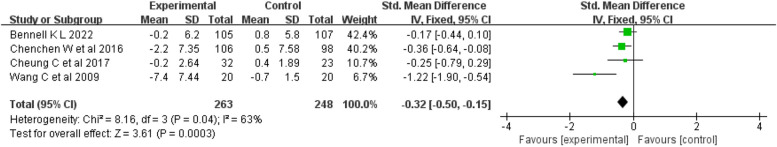
Forest plots showing standardized mean difference of change on the depression between Mind–body exercise group and a control/comparison group

Depression: Four studies [21, 22, 24, 30] were included in the depression analysis, for a total sample of 511 people. The meta-analysis indicated substantial heterogeneity among the studies (I^2^ = 63%, *p* = 0.04). The results indicated (Fig. 8) that mind–body exercise was significantly better than was the control condition at improving depression (fixed-effects model, SMD = -0.32; 95% CI: -0.50, -0.15; *p* = 0.0003).

### Effect of mind–body exercise on 6-MWT and TUG times

The 6-MWT and TUG times were measured using the same methods in all the included studies, with meters (of distance) and stopwatches, respectively. The WMDs and 95% CIs were calculated to determine the effect size.

**Fig. 9:**
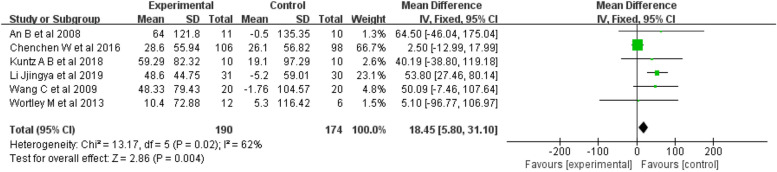
Forest plots showing standardized mean difference of change on the 6-MWT between Mind–body exercise group and a control/comparison group

6-MWT: Six studies [19, 22, 26, 28, 30, 31] were included in the 6-MWT analysis, for a total sample of 364 people. The meta-analysis indicated substantial heterogeneity among the studies (I^2^ = 62%, *p* = 0.02). The results indicated (Fig. 9) that mind–body exercise was significantly better than was the control condition at increasing 6-MWT time (fixed-effects model, WMD = 18.45; 95% CI: 5.8, 31.1; *p* = 0.004).

**Fig. 10:**
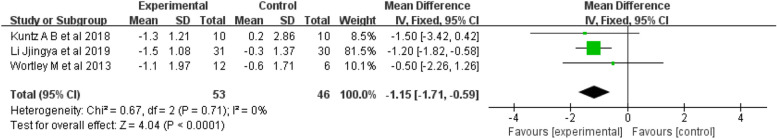
Forest plots showing standardized mean difference of change on the TUG between Mind–body exercise group and a control/comparison group

TUG time: Three studies [26, 28, 31] were included in the TUG analysis, for a total sample of 99 people. The meta-analysis indicated no heterogeneity among the studies (I^2^ = 0%, *p* = 0.71). The results indicated (Fig. 10) that mind–body exercise was significantly better than was the control condition at reducing TUG time (fixed-effects model, WMD = -1.15; 95% CI: -1.71, -0.59; *p* < 0.0001).

### Sensitivity analysis

**Fig. 11:**
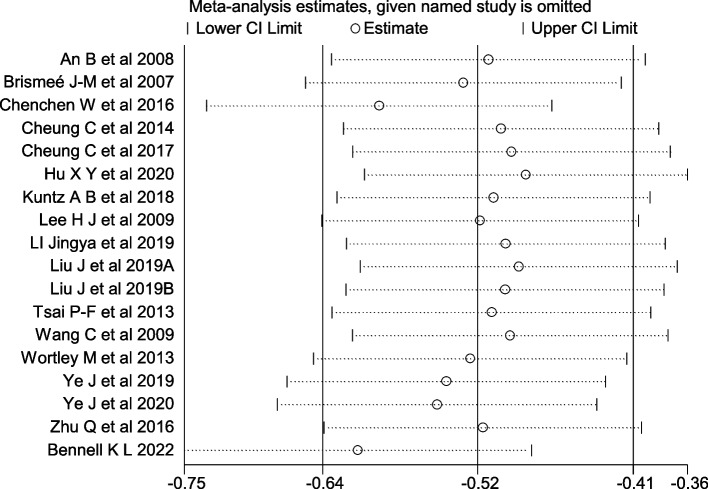
Sensitivity Analysis Plot of Pain

**Fig. 12:**
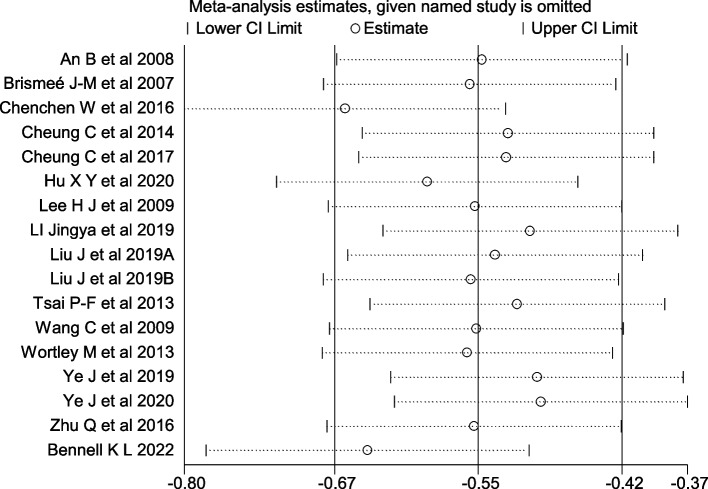
Sensitivity Analysis Plot of Stiffness

**Fig. 13:**
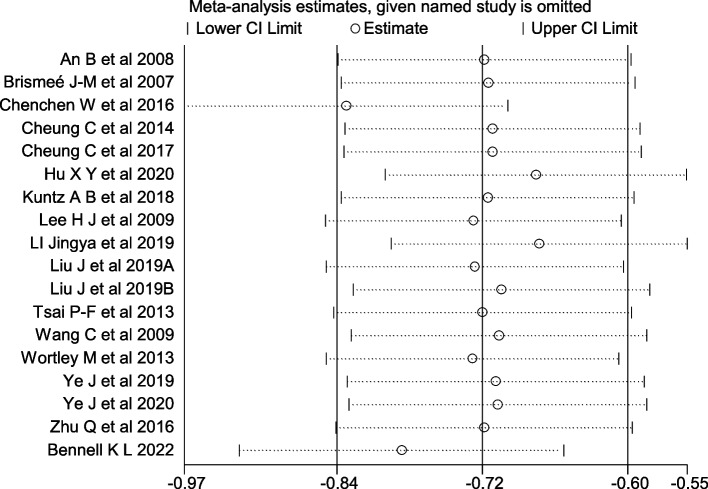
Sensitivity Analysis Plot of Physical function

Sensitivity analysis was performed for variables with substantial heterogeneity using Stata 14.0. The results are shown in Figs. 11, 12 and 13. The included pain, stiffness and physical function variables had good sensitivity, as exclusion of any individual experiment did not lead to significant changes in the total effect size. According to the sensitivity analysis, the pooled effect of mind–body exercise on improving pain, stiffness and physical function was robust.

### Evaluation of publication bias

Egger's test was used to detect publication bias in experiments with substantial heterogeneity. The test result for the effect of mind–body exercise on pain was t = -2.69 (95% CI: -4.634, -0.552; *P* = 0.016). Egger’s test of the effect of mind–body exercise on stiffness yielded a t = -2.93 (95% CI = -6.417, -1.01; *P* = 0.01). Egger’s test of the effect of mind–body exercise on physical function intervention yielded a t = -2.59 (95% CI: -4.133, -0.415; *P* = 0.023). The results suggest that there was publication bias in the intervention effects of mind–body exercise on pain, stiffness, and physical function.

## Discussion

### Overall findings

Mind–body exercise, described as low-intensity exercise that enhances body–mind coordination and awareness through exercise, such as by controlling movement and concentration [35] and focusing on interactions among the mind, brain, behavior, and body [36], has been used to treat a variety of chronic pain disorders [37–39]. This form of exercise is considered suitable for middle-aged and elderly individuals [40, 41]. There are many advantages to using mind–body exercise as an adjunctive treatment for patients with KOA compared with first-line treatments (medications, physical therapy, intra-articular injections). There are few known adverse events associated with this type of exercise, which is particularly important for older patients who are prone to drug side effects and potential drug‒drug interactions. Compared to traditional exercises, which target muscle strength and the cardiovascular and respiratory systems, mind–body exercise has additional physiological, psychological, and clinical effects [42]. Fogaca LZ et al. [43] suggested mind–body exercises for different disease fields based on an evidence map, providing a reference for patients and researchers. The present meta-analysis combined data from three types of mind–body exercises: tai chi, yoga, and baduanjin. Eighteen RCTs (17 articles) with a sample size of 1,122 people were included. The results showed that mind–body exercise can effectively improve pain, stiffness, physical function, mental health, depression, and motor ability.

Selfe TK et al. [44] conducted a systematic review of the effects of tai chi, yoga, and baduanjin on pain and physical function in patients with KOA; however, they did not perform a meta-analysis. Our study analyzed additional outcome indicators (pain, physical function, stiffness, mental health, depression, 6-MWT time, and TUG time) and included additional RCTs (18). Our meta-analysis showed that, compared with the control group, mind–body exercise significantly improved pain (SMD = -0.65; 95% CI: -0.87, -0.42; *p* < 0.00001), stiffness (SMD = -0.75; 95% CI: -1.05, -0.45; *p* < 0.00001), and physical function (SMD = -0.82; 95% CI: -1.03, -0.62; *p* < 0.00001). Goh et al. [45] compared the effects of different exercise interventions on the physical function, pain, and quality of life of patients with knee and hip osteoarthritis. Mind–body exercise and aerobic exercise seemed to yield the greatest improvements in pain and physical function. They found that mind–body exercise and aerobic exercise were equally effective at reducing pain.

Because of the high heterogeneity, subgroup analyses based on exercise type, duration, frequency, sex, and region were conducted. In terms of improving pain, stiffness, and physical function, the source of heterogeneity might be attributed to the duration and frequency of mind–body exercises. In the included studies, the duration of mind–body exercises ranged from 8 to 24 weeks, and the frequency was 2–5 times per week. Mind–body exercise twice a week did not significantly improve stiffness. With a duration of more than 12 weeks, the effects of mind–body exercise on pain, stiffness and physical function were more significant.

In addition, the subgroup analysis showed that tai chi, yoga, and baduanjin had consistent beneficial effects on stiffness and physical function. These findings are consistent with previous meta-analyses involving one or two types of mind–body exercises [5, 46–49]. In terms of pain, the present study showed that the effects of tai chi and yoga on pain improvement were significant, while the effect of baduanjin was not significant, which is consistent with the subgroup analysis of Li et al. [5]. A review of the included data revealed some inconsistent results; specifically, Ye et al. [32, 33] reported that a 12-week baduanjin intervention did not significantly improve pain, which is inconsistent with the results of Liu et al. [18]. Differences in interventions (such as in the frequency and duration of exercise) and in assessment scales among the studies may have affected the research findings. Few studies have evaluated the use of a baduanjin intervention in KOA patients, and additional RCTs are needed for further evaluation.

Depression is associated with pain, worse physical function, and structural disease progression in patients with osteoarthritis [50]. One-fifth of adults with osteoarthritis suffer from depression in addition to chronic pain [51]. Available evidence [52, 53] suggests a bidirectional relationship between pain and depression; that is, each affects the severity of the other. However, depression is often overlooked—in particular, overt osteoarthritis symptoms mask less apparent depression symptoms—limiting its diagnosis [54]. Future treatments should focus on both pain and depression in KOA patients. Kroenke et al. [55] confirmed that simultaneous treatment of depression and pain can improve pain and physical function in patients. Rathbun et al. [56] explained why a single treatment strategy is not effective for osteoarthritis patients with depression. Among KOA patients, depressive symptoms have been associated with poorer future physical function, but the relationship is partly mediated by pain (approximately one-fifth), and the magnitude of the effect decreases with increasing depressive symptoms. The results of the present study showed that in addition to reducing pain, mind–body exercise also significantly improved depression in patients, which may explain why mind–body exercise yields substantial benefits.

Another notable effect of mind–body exercise is the improved motor ability of KOA patients. The meta-analysis showed that, compared with the control condition, mind–body exercise significantly increased the 6-MWT time (18.45; 95% CI: 5.80, 31.10; *p* = 0.004) and significantly decreased the TUG time (-1.15; 95% CI: -1.71, -0.59; *p* < 0.0001). These findings are consistent with those of the meta-analysis on tai chi by Hu et al. [46]. Shimizu H et al. reported that patients with early KOA took longer to complete the TUG test [57]. In a separate study on knee osteoarthritis, ALGHADIR et al. reported minimal detectable change (MDC) values for the TUG test of 1.14 s [58].

At present, the specific mechanism underlying the effect of mind–body exercise on KOA patients has not been fully elucidated. Preliminary evidence suggests that mind–body exercise may exert therapeutic effects by improving joint proprioception and inducing central nervous system plasticity. Proprioception is necessary for preventing excessive movement and maintaining postural stability and motor coordination, which are potentially important for preventing joint injury. KOA patients exhibit impaired proprioceptive (positional and motor sense) accuracy, but this appears to be the result of structural degeneration rather than an early risk factor for KOA onset [59]. Multiple RCTs have shown that mind–body exercise positively affects knee proprioception [32, 60, 61].

The dorsolateral prefrontal cortex (DLPFC) plays an important role in pain regulation. After a 12-week intervention consisting of tai chi and baduanjin, KOA patients showed decreased resting-state functional connectivity between the DLPFC and supplementary motor areas as well as enhanced resting-state functional connectivity between the DLPFC and anterior cingulate cortex [62]. In addition, tai chi significantly increased the gray matter volume in the auxiliary motor areas. Shen et al. explored the relationships of brain functional connectivity with pain and physical function in postmenopausal women with KOA after an 8-week tai chi intervention [63]. The study revealed a moderate-to-high correlation between postintervention changes in connectivity between the amygdala and medial prefrontal cortex and improvements in pain and physical function, suggesting that mind–body exercise may modulate pain and physical function by directly affecting the cerebral cortex.

### Limitations

This study has several limitations. First, the included studies were limited to those published in English or Chinese, which may have led to incompleteness bias. Second, this study analyzed only data collected immediately after the intervention; thus, it lacked analysis of long-term effects. Third, there are obvious shortcomings in the methods used in these studies. For example, more than 70% of the studies had a sample size of less than 30, most studies were single-blinded (evaluator), and some studies lacked allocation concealment, which may have affected the study results. Finally, mind–body exercise intervention programs vary greatly, and this paper does not provide suggestions on the optimal exercise scheme (such as exercise frequency, exercise timing, or exercise duration).

## Conclusions

Mind–body exercise can effectively improve pain, stiffness, physical function, mental health, depression, and motor ability in KOA patients. After combining these results with the low risk of adverse events in the included studies, we concluded that mind–body exercise is a safe and effective KOA intervention. Given the methodological limitations of the included studies, additional high-quality evidence is needed to support the conclusions of this study.

## Data Availability

The data are available in this published article and can be procured from the corresponding author upon request.

## Abbreviations

KOA: Knee osteoarthritis
RCTs: Randomized controlled trials
KOOS: Knee Injury and Osteoarthritis Outcome Score
6-MWT: Six-minute walk test
WOMAC: Western Ontario and McMaster Universities Osteoarthritis Index
TUG: Timed up and go
DLPFC: Dorsolateral prefrontal cortex

## References

[1] Park HM, Kim HS, Lee YJ (2020). Knee osteoarthritis and its association with mental health and health-related quality of life: a nationwide cross-sectional study. Geriatr Gerontol Int.

[2] Brophy RH, Fillingham YA. AAOS Clinical Practice Guideline Summary: Management of Osteoarthritis of the Knee (Nonarthroplasty), Third Edition. J Am Acad Orthopaedic Surgeons. 2022;30(9):e721-e729. 10.5435/JAAOS-D-21-01233 35383651

[3] McAlindon TE, Bannuru RR, Sullivan MC, Arden NK, Berenbaum F, Bierma-Zeinstra SM (2014). OARSI guidelines for the non-surgical management of knee osteoarthritis. Osteoarthritis Cartilage.

[4] Kolasinski SL, Neogi T, Hochberg MC, Oatis C, Guyatt G, Block J (2020). 2019 American College of Rheumatology/Arthritis Foundation Guideline for the Management of Osteoarthritis of the Hand, Hip, and Knee. Arthritis Care Res.

[5] Li R, Chen H, Feng J, Xiao Y, Zhang H, Lam CW (2020). Effectiveness of traditional Chinese exercise for symptoms of knee osteoarthritis: a systematic review and meta-analysis of randomized controlled trials. Int J Environ Res Public Health.

[6] Weeks J (2016). The New USA NIH Strategic Plan for Complementary and Integrative Health: Interview with Josephine Briggs, md. Alternative Complementary Medicine.

[7] Clarke TC, Black LI, Stussman BJ, Barnes PM, Nahin RL (2015). Trends in the use of complementary health approaches among adults: United States, 2002–2012. Natl Health Stat Report.

[8] Bower JE, Irwin MR (2016). Mind-body therapies and control of inflammatory biology: a descriptive review. Brain Behav Immun.

[9] Bo A, Mao W, Lindsey MA (2017). Effects of mind-body interventions on depressive symptoms among older Chinese adults: a systematic review and meta-analysis. Int J Geriatr Psychiatry.

[10] Reychler G, Poncin W, Montigny S, Luts A, Caty G, Pieters T (2019). Efficacy of yoga, tai chi and qi gong on the main symptoms of chronic obstructive pulmonary disease: a systematic review. Respir Med Res.

[11] Li Z, Liu S, Wang L, Smith L (2019). Mind-body exercise for anxiety and depression in COPD patients: a systematic review and meta-analysis. Int J Environ Res.

[12] Wen YR, Shi J, Wang YF, Lin YY, Hu ZY, Lin YT (2022). Are mind-body exercise beneficial for treating pain, function, and quality of life in middle-aged and old people with chronic pain? A systematic review and meta-analysis. Front Aging Neurosci.

[13] Eyre HA, Acevedo B, Yang H, Siddarth P, Van Dyk K, Ercoli L (2016). Changes in neural connectivity and memory following a yoga intervention for older adults: a pilot study. J Alzheimer's Dis.

[14] Farhang M, Miranda-Castillo C, Rubio M, Furtado G (2019). Impact of mind-body interventions in older adults with mild cognitive impairment: a systematic review. Int Psychogeriatr.

[15] Sungkarat S, Boripuntakul S, Chattipakorn N, Watcharasaksilp K, Lord SR (2017). Effects of Tai Chi on cognition and fall risk in older adults with mild cognitive impairment: a randomized controlled trial. J Am Geriatr Soc.

[16] Kim SH, Schneider SM, Kravitz L, Mermier C, Burge MR (2013). Mind-body practices for posttraumatic stress disorder. J Investig Med.

[17] Cumpston M, Li T, Page MJ, Welch JCVA, Higgins JP, Thomas J. Updated guidance for trusted systematic reviews a new edition of the Cochrane Handbook for Systematic Reviews of Interventions. Cochrane Database Syst Rev. 2019;10:ED000142. 10.1002/14651858.ED000142 PMC10284251 31643080

[18] Liu J, Chen L, Chen X, Hu K, Tu Y, Lin M (2019). Modulatory effects of different exercise modalities on the functional connectivity of the periaqueductal grey and ventral tegmental area in patients with knee osteoarthritis: a randomised multimodal magnetic resonance imaging study. Br J Anaesth.

[19] An B, Dai K, Zhu Z, Wang Y, Hao Y, Tang T (2008). Baduanjin alleviates the symptoms of knee osteoarthritis. Alternative Complementary Medicine.

[20] Brisme´e J-M, Paige RL, Chyu M-C, Boatright JD. Group and home-based tai chi in elderly subjects with knee osteoarthritis: a randomized controlled trial. Clin Rehabil. 2007; 21(2):99–111. 10.1177/0269215506070505 17264104

[21] Bennell KL, Schwartz S, Teo PL, Hawkins S, Mackenzie D, McManus F (2022). Effectiveness of an unsupervised online yoga program on pain and function in people with knee osteoarthritis: a randomized clinical trial. Ann Intern Med.

[22] Wang C, Schmid CH, Iversen MD, Harvey WF, Fielding RA, Driban JB (2016). Comparative effectiveness of tai chi versus physical therapy for knee osteoarthritis: a randomized trial. Ann Intern Med.

[23] Cheung C, Wyman JF, Resnick B, Savik K (2014). Yoga for managing knee osteoarthritis in older women: a pilot randomized controlled trial. BMC Complement Altern Med.

[24] Cheung C, Wyman JF, Bronas U, McCarthy T, Rudser K, Mathiason MA (2017). Managing knee osteoarthritis with yoga or aerobic/strengthening exercise programs in older adults: a pilot randomized controlled trial. Rheumatol Int.

[25] Hu X, Lai Z, Wang L (2020). Effects of Taichi exercise on knee and ankle proprioception among individuals with knee osteoarthritis. Res Sports Med.

[26] Kuntz AB, Chopp-Hurley JN, Brenneman EC, Karampatos S, Wiebenga EG, Adachi JD (2018). Efficacy of a biomechanically-based yoga exercise program in knee osteoarthritis: a randomized controlled trial. PLoS ONE.

[27] Lee H-J, Park H-J, Chae Y, Kim S-Y (2009). Tai Chi Qigong for the quality of life of patients with knee osteoarthritis a pilot, randomized, waiting list controlled trial. Clin Rehabil.

[28] Jingya L, Liang C (2019). The effect of Taichi and resistance training on osteoarthritis symptoms of the elderly and the exercise capacity. Chin J Rehabil Med.

[29] Tsai PF, Chang JY, Beck C, Kuo YF, Keefe FJ (2013). A pilot cluster-randomized trial of a 20-week Tai Chi program in elders with cognitive impairment and osteoarthritic knee: effects on pain and other health outcomes. J Pain Symptom Manage.

[30] Wang C, Schmid CH, Hibberd PL, Kalish R, Roubenoff R, Rones R (2009). Tai Chi is effective in treating knee osteoarthritis: a randomized controlled trial. Arthritis Rheum.

[31] Wortley M, Zhang S, Paquette M, Byrd E, Baumgartner L, Klipple G (2013). Effects of resistance and Tai Ji training on mobility and symptoms in knee osteoarthritis patients. J Sport Health Sci.

[32] Ye J, Simpson MW, Liu Y, Lin W, Zhong W, Cai S (2019). The effects of Baduanjin qigong on postural stability, proprioception, and symptoms of patients with knee osteoarthritis: a randomized controlled trial. Front Med.

[33] Ye J, Zheng Q, Zou L, Yu Q, Veronese N, Grabovac I (2020). Mindful Exercise (Baduanjin) as an Adjuvant Treatment for Older Adults (60 Years Old and Over) of Knee Osteoarthritis: A Randomized Controlled Trial. Evidence-Based Complementary Alternative Medicine.

[34] Zhu Q, Huang L, Wu X, Wang L, Zhang Y, Fang M (2016). Effects of Tai Ji Quan training on gait kinematics in older Chinese women with knee osteoarthritis: a randomized controlled trial. J Sport Health Sci.

[35] Kwok JY, Choi KC, Chan HY (2016). Effects of mind-body exercises on the physiological and psychosocial well-being of individuals with Parkinson's disease: a systematic review and meta-analysis. Complement Ther Med.

[36] Augsburg JB, Dar MI, Wood K, Rasmussen TB, Risom SS (2022). A systematic literature review of the effect of mind-body interventions on mental health among patients with atrial fibrillation. Mental Health Prev.

[37] Liu J, Yeung A, Xiao T, Tian X, Kong Z, Zou L (2019). Chen-Style Tai Chi for Individuals (Aged 50 Years Old or Above) with Chronic Non-Specific Low Back Pain: A Randomized Controlled Trial. Int J Environ Res Public Health.

[38] Wang C, Schmid CH, Fielding RA, Harvey WF, Reid KF, Price LL (2018). Effect of tai chi versus aerobic exercise for fibromyalgia: comparative effectiveness randomized controlled trial. BMJ.

[39] Wang J-Y, Guo H, Tang L, Meng J, Hu L-Y. Case-control study on regular Ba Duan Jin practice for patients with chronic neck pain. Int J Nurs Sci. 2014;1(4):360–366.

[40] Reid MC, Eccleston C, Pillemer K (2015). Management of chronic pain in older adults. BMJ.

[41] Siu PM, Yu AP, Chin EC, Yu DS, Hui SS, Woo J (2021). Effects of Tai Chi or Conventional Exercise on Central Obesity in Middle-Aged and Older Adults : A Three-Group Randomized Controlled Trial. Ann Intern Med.

[42] Brosseau L, Taki J, Desjardins B, Thevenot O, Fransen M, Wells GA, et al. The Ottawa panel clinical practice guidelines for the management of knee osteoarthritis. Part one: introduction, and mind-body exercise programs. Clin Rehabil. 2017; 31(5):582–595. 10.1177/0269215517691083 28183188

[43] Fogaca LZ, Portella CFS, Ghelman R, Abdala CVM, Schveitzer MC (2021). Mind-body therapies from traditional Chinese medicine: evidence map. Front Public Health.

[44] Selfe TK, Innes KE (2009). Mind-body therapies and osteoarthritis of the knee. Curr Rheumatol Rev.

[45] Goh SL, Persson MSM, Stocks J, Hou Y, Welton NJ, Lin J (2019). Relative efficacy of different exercises for pain, function, performance and quality of life in knee and hip osteoarthritis: systematic review and network meta-analysis. Sports Med.

[46] Hu L, Wang Y, Liu X, Ji X, Ma Y, Man S (2021). Tai Chi exercise can ameliorate physical and mental health of patients with knee osteoarthritis: systematic review and meta-analysis. Clin Rehabil.

[47] Zeng ZP, Liu YB, Fang J, Liu Y, Luo J, Yang M (2020). Effects of Baduanjin exercise for knee osteoarthritis: a systematic review and meta-analysis. Complement Ther Med.

[48] Zhang Y, Huang L, Su Y, Zhan Z, Li Y, Lai X (2017). The effects of traditional Chinese exercise in treating knee osteoarthritis: a systematic review and meta-analysis. PLoS ONE.

[49] Wang Y, Lu S, Wang R, Jiang P, Rao F, Wang B (2018). Integrative effect of yoga practice in patients with knee arthritis: a PRISMA-compliant meta-analysis. Medicine.

[50] Rathbun AM, Shardell MD, Ryan AS, Yau MS, Gallo JJ, Schuler MS (2020). Association between disease progression and depression onset in persons with radiographic knee osteoarthritis. Rheumatology.

[51] Fonseca-Rodrigues D, Rodrigues A, Martins T, Pinto J, Amorim D, Almeida A (2021). Correlation between pain severity and levels of anxiety and depression in osteoarthritis patients: a systematic review and meta-analysis. Rheumatology.

[52] Parmelee PA, Behrens EA, Costlow Hill K, Cox BS, DeCaro JA, Keefe FJ (2022). Momentary associations of osteoarthritis pain and affect: depression as moderator. J Gerontol B Psychol Sci Soc Sci.

[53] Riddle DL, Kong X, Fitzgerald GK (2011). Psychological health impact on 2-year changes in pain and function in persons with knee pain: data from the Osteoarthritis Initiative. Osteoarthritis Cartilage.

[54] Wang ST, Ni GX (2022). Depression in Osteoarthritis: Current Understanding. Neuropsychiatr Dis Treat.

[55] Kroenke K, Bair MJ, Damush TM, Wu J, Hoke S, Sutherland J (2009). Optimized antidepressant therapy and pain self-management in primary care patients with depression and musculoskeletal pain: a randomized controlled trial. JAMA.

[56] Rathbun AM, Shardell MD, Stuart EA, Yau MS, Gallo JJ, Schuler MS (2018). Pain severity as a mediator of the association between depressive symptoms and physical performance in knee osteoarthritis. Osteoarthritis Cartilage.

[57] Shimizu H, Shimoura K, Iijima H, Suzuki Y, Aoyama T (2022). Functional manifestations of early knee osteoarthritis: a systematic review and meta-analysis. Clin Rheumatol.

[58] Alghadir A, Anwer S, Brismee JM (2015). The reliability and minimal detectable change of Timed Up and Go test in individuals with grade 1–3 knee osteoarthritis. BMC Musculoskelet Disord.

[59] Baert IA, Mahmoudian A, Nieuwenhuys A, Jonkers I, Staes F, Luyten FP (2013). Proprioceptive accuracy in women with early and established knee osteoarthritis and its relation to functional ability, postural control, and muscle strength. Clin Rheumatol.

[60] Schmid A, McAlindon T, H C, Wang C. The Influence of Tai Chi Exercise on Proprioception in Patients with Knee Osteoarthritis: Results from a Pilot Randomized Controlled Trial. Int J Integr Med. 2013;1:37. 10.5772/57137 PMC5578627 28868082

[61] Qingguang Z, Lingyan H, Jingxian L, Lijuan M, Yunya Z (2017). Effect of Taijiquan practice versus wellness education on knee proprioception in patients with knee osteoarthritis a randomized controlled trial. J Tradit Chin Med.

[62] Liu J, Chen L, Tu Y, Chen X, Hu K, Tu Y (2019). Different exercise modalities relieve pain syndrome in patients with knee osteoarthritis and modulate the dorsolateral prefrontal cortex: a multiple mode MRI study. Brain Behav Immun.

[63] Shen CL, Watkins BA, Kahathuduwa C, Chyu MC, Zabet-Moghaddam M, Elmassry MM (2021). Tai Chi improves brain functional connectivity and plasma lysophosphatidylcholines in postmenopausal women with knee osteoarthritis: an exploratory pilot study. Front Med.

